# Comparison of clinical features and outcomes between patients with early and delayed lupus nephritis

**DOI:** 10.1186/s12882-020-01915-5

**Published:** 2020-07-07

**Authors:** Sung Soo Ahn, Juyoung Yoo, Seung Min Jung, Jason Jungsik Song, Yong-Beom Park, Sang-Won Lee

**Affiliations:** 1 Division of Rheumatology, Department of Internal Medicine, Yonsei University College of Medicine, 50-1 Yonsei-ro, Seodaemun-gu, Seoul, 03722 Republic of Korea; 2 Institute for Immunology and Immunological Diseases, Yonsei University College of Medicine, Seoul, Republic of Korea

**Keywords:** Lupus nephritis, Early, Delayed, Prognosis, Predictor

## Abstract

**Background:**

Lupus nephritis is associated with increased risk of end-stage renal disease (ESRD) and all-cause mortality. We evaluated the clinical features and outcomes of patients with early and delayed lupus nephritis.

**Methods:**

The medical records of 171 patients who met the 1997 revised classification criteria for systemic lupus erythematosus (SLE) with pathologic confirmation of lupus nephritis were reviewed. Early lupus nephritis was defined when lupus nephritis was histopathologically confirmed as the first clinical manifestation of SLE, whereas delayed lupus nephritis was defined as lupus nephritis that was identified after the diagnosis of SLE. Clinical and laboratory data, as well as kidney histopathology and medication usage were investigated. Kaplan-Meier and Cox-proportional hazard analysis was performed to compare the outcomes of early and delayed lupus nephritis and evaluate factors associated with ESRD and all-cause mortality.

**Results:**

Patients with early lupus nephritis had higher disease activity (median non-renal SLE disease activity index-2000, 6.0 vs. 4.0; *p* < 0.001) and more frequent skin rash, oral ulcer and serositis; however, the proportion of patients with higher renal chronicity index was greater in the delayed lupus nephritis group (*p* = 0.007). Nevertheless, no difference was found regarding ESRD and all-cause mortality between the groups. In Cox-proportional hazard analysis, C-reactive protein level, creatinine level and chronicity index were factors associated with ESRD, while age and haemoglobin level were associated with all-cause mortality.

**Conclusions:**

In conclusion, clinical outcomes of early and delayed lupus nephritis are not significantly different. Rigorous adherence to current treatment recommendations is essential for the treatment of lupus nephritis.

## Background

Systemic lupus erythematosus (SLE) is an idiopathic inflammatory disease characterized by multiple organ injury as a result of autoantibody formation [1]. Lupus nephritis is one of the most serious systemic complication of SLE that affects up to 60–70% of patients with SLE during their lifetime [2]. Lupus nephritis is classified into 6 different subtypes based on the pathologic findings, with class III and class IV (proliferative) lupus nephritis being the most aggressive form [3]. The treatment of proliferative lupus nephritis consists of induction therapy, followed by maintenance therapy to achieve remission [4]. However, the prognosis of patients with lupus nephritis remains unfavourable even when aggressive treatment strategies are implemented, mainly because of a significantly increased risk of end-stage renal disease (ESRD) and all-cause mortality [5]. Accordingly, the treatment guidelines for SLE recommend prudent monitoring of patients for the development of lupus nephritis [6]. In general, lupus nephritis primarily occurs at the time of or within the first year of SLE diagnosis [7]. On the contrary, the frequency of lupus nephritis occurrence gradually decreases with time; the onset of lupus nephritis is reported to be uncommon after 5 years from SLE diagnosis [8].

Most patients with SLE are treated based on the organ involved, and immunosuppressive agents, such as glucocorticoids and hydroxychloroquine, are the most widely used drugs for the management of SLE [9]. A considerable number of patients who had been diagnosed with SLE before lupus nephritis (delayed lupus nephritis), may have been exposed to glucocorticoids and/or immunosuppressive drugs, while most patients diagnosed with lupus nephritis as the first complication of SLE (early lupus nephritis) may be glucocorticoid and/or immunosuppressive drug-naive. Therefore, it is theoretically assumed that the clinical features at the time of kidney biopsy and the prognosis may differ between early and delayed lupus nephritis proportionally to the interval between the diagnosis of SLE and lupus nephritis; however, the data from the literature regarding this subject are scarce [10]. Therefore, the objectives of this study were 1) to compare the clinical features of early and delayed lupus nephritis at the time of kidney biopsy and its prognosis and 2) to investigate factors associated with ESRD and all-cause mortality during the follow-up period.

## Methods

### Patient selection

The records of patients diagnosed with lupus nephritis by kidney biopsy between January 2006 and July 2018 were retrospectively reviewed. The following patients were included: i) patients with histological findings compatible with 2003 International Society of Nephrology/ Renal Pathology Society (ISN/RPS) classification criteria for lupus nephritis [3]; ii) patients who met the 1997 revised American College of Rheumatology (ACR) classification criteria for SLE [11, 12]; iii) patients who were not diagnosed with lupus nephritis prior to pathological confirmation. In case of patients with repeated histopathological results, the first result was used in this study. Finally, 171 patients with lupus nephritis were included in this study. This study was approved by the Institutional Review Board of Severance Hospital, and the need for written informed consent was waived, as this was a retrospective study (4–2018-1083).

### Evaluation of clinical and laboratory data and medications

All clinical and laboratory data were assessed at the time of kidney biopsy. Kidney biopsy was performed in the patients when the amount of proteinuria was greater than 1 g per 24 h (either in 24 h urine or spot urine protein/creatinine (P/Cr) ratio) in the absence of alternative causes. The demographic data included age, sex and disease duration after the diagnosis of SLE. The SLE specific variables included non-renal SLE disease activity index-2000 (SLEDAI-2 K), complement (C)3, C4, anti-dsDNA and spot urine P/Cr ratio [13]. Laboratory data collected were white blood cell count, platelet count, lymphocyte count, hemoglobin level, erythrocyte sedimentation rate (ESR), C-reactive protein (CRP), creatinine level and albumin level. Investigated clinical manifestations comprised the components of 1997 ACR classification criteria, including skin rash, photosensitivity, oral ulcer, arthritis, serositis and neurologic, hematologic and immunologic disorder [11]. The number of patients with hypertension, diabetes mellitus and dyslipidaemia prior to the diagnosis of lupus nephritis was counted based on international classification of diseases-10 and Korean drug utilization review (DUR) system. For kidney histopathology data, lupus nephritis class was determined according to the 2003 ISN/RPS criteria, and the activity and chronicity index were calculated based on the scoring system from the National Institutes of Health [3, 14]. Medications that were used prior and after the diagnosis of lupus nephritis were defined as those for achieving or maintaining the remission of SLE and were searched by using the Korean DUR system. In addition, the adverse effects of glucocorticoid and immunosuppressive agents and the cumulative dosage of glucocorticoid was calculated by using the Hospital’s electronic medical record system.

### Definition of early and delayed lupus nephritis and clinical outcomes

In this study, early lupus nephritis was defined when lupus nephritis was histopathologically confirmed as the first clinical manifestation of SLE, whereas delayed lupus nephritis was defined as lupus nephritis that was identified after the diagnosis of SLE. In addition, ESRD was defined as an impairment of renal function that required dialysis, while all-cause mortality as death for any reason during the follow-up. The follow-up duration was determined as the gap-time from the diagnosis of lupus nephritis to the last visit for the survived patients, to death for the deceased patients, and to the initiation of dialysis for the patients with ESRD. Composite outcome was defined as either the presence of ESRD and/or all-cause mortality.

### Statistical analysis

All statistical analyses were performed using MedCalc statistical software version 18.11.3 (MedCalc Software, Ostend, Belgium). Continuous and categorical variables were represented as median (interquartile ranges) and frequencies (percentages). Comparison of continuous variables was performed by Mann-Whitney U test and categorical variables were compared using chi-square, chi-square for trend, or the Fisher’s exact test as appropriate. Kaplan-Meier analysis with the log-rank test was used to compare the clinical outcomes of early and delayed lupus nephritis. Multivariate Cox-proportional hazard analysis using statistically significant variables in univariate analysis was used to evaluate factors associated with ESRD and all-cause mortality. In all analysis, a two-tailed *p* < 0.05 was considered statistically significant.

## Results

### Comparison of patient characteristics

The baseline characteristics of patients are shown in Table 1. The median age was 36.0 years, 153 (89.5%) patients were women and the median follow-up duration was 57.1 months. As we divided our patients into early and delayed lupus nephritis, 106 (62.0%) and 65 (38.0%) patients were classified as having early and delayed lupus nephritis. The median disease duration of delayed lupus nephritis group was 52.6 months, and there was no difference in the follow-up duration between the groups after the diagnosis of lupus nephritis was established. Patients with early nephritis had higher non-renal SLEDAI-2 K and ESR, but lower anti-dsDNA, WBC count and albumin levels compared to those with delayed lupus nephritis. Regarding clinical manifestations, patients with early lupus nephritis had a higher incidence of skin rash, oral ulcer and serositis compared to those with delayed lupus nephritis. In kidney histopathology data, no difference was found in lupus nephritis classes and activity index, but the proportion of patients with higher chronicity index was significantly greater in the delayed lupus nephritis group (*p* = 0.007). Glucocorticoids were the most frequently selected immunosuppressive agents that were used in the delayed lupus nephritis group prior to the diagnosis of lupus nephritis, followed by hydroxychloroquine and azathioprine (Table 2). Medications that were used after the diagnosis of lupus nephritis were not significantly different between the groups, except for cyclophosphamide that was more frequently used in patients with early lupus nephritis than in those with delayed lupus nephritis (39.6% vs. 23.1%, *p* = 0.026, Table 3). When we investigated the adverse effects of glucocorticoid and immunosuppressive agents, systemic effects were the most common, followed by infections in both early and delayed lupus nephritis group. There was no significant difference in adverse effects between the groups, only except that myalgia was more common in patients with early lupus nephritis (Supplementary Table 1).

**Table 1:** Baseline characteristics of patients with early and delayed lupus nephritis

Variables	Total (*n* = 171)	Patients with early lupus nephritis (*n* = 106)	Patients with delayed lupus nephritis (*n* = 65)	*p*-value
**Demographic data**				
Age, years	36.0 (26.3–46.0)	36.0 (25.0–48.0)	36.0 (29.0–44.0)	0.903
Female sex, n (%)	153 (89.5)	91 (85.8)	62 (95.4)	0.070
Disease duration, months	n/a	n/a	52.6 (22.0–118.8)	< 0.001
Follow-up duration, months	57.1 (17.5–90.8)	40.9 (14.2–91.3)	67.6 (27.0–88.9)	0.119
**SLE specific variables**				
Non-renal SLEDAI-2 K	5.0 (4.0–7.0)	6.0 (5.0–8.0)	4.0 (4.0–6.0)	< 0.001
Complement 3, mg/dL	42.7 (28.7–66.4)	40.3 (26.2–67.3)	46.4 (34.9–64.5)	0.129
Low complement 3, n (%)	154 (90.1)	95 (89.6)	59 (90.8)	0.808
Complement 4, mg/dL	5.6 (3.0–12.5)	5.3 (3.0–12.5)	6.2 (3.5–12.3)	0.623
Low complement 4, n (%)	114 (66.7)	73 (68.9)	41 (63.1)	0.437
Anti-dsDNA (IU/mL)	218.4 (32.3–379.0)	160.0 (10.0–379.0)	300.0 (143.3–379.0)	0.009
Elevated anti-dsDNA, n (%)	141 (82.5)	83 (78.3)	58 (89.2)	0.069
Spot urine P/Cr ratio	2.9 (1.5–6.0)	3.4 (1.5–6.5)	2.3 (1.5–4.2)	0.123
**Laboratory data**				
WBC count (/μL)	4400.0 (3147.5–6145.0)	3925.0 (3030.5630.0)	4950.0 (3947.5–6727.5)	0.018
Platelet count (× 1000/μL)	203.0 (136.3–247.8)	200.0 (113.0–240.0)	213.0 (152.8–253.3)	0.126
Hemoglobin (g/dL)	10.4 (9.1–11.7)	10.2 (8.9–11.5)	10.9 (9.5–11.9)	0.189
Lymphocyte count (/μL)	860.0 (580.0–1227.5)	840.0 (550.0–1250.0)	900.0 (610.-1222.5)	0.999
ESR (mm/hr)	53.0 (28.5–76.0)	57.0 (35.0–84.0)	41.0 (24.8–68.8)	0.013
CRP (mg/L)	2.5 (1.0–8.9)	3.0 (1.0–9.6)	1.9 (1.0–4.9)	0.074
Cr (mg/dL)	0.8 (0.6–1.1)	0.8 (0.6–1.1)	0.8 (0.6–1.0)	0.434
Albumin (g/dL)	2.9 (2.2–3.4)	2.6 (2.1–3.3)	3.2 (2.6–3.4)	0.002
**Clinical manifestations**				
Skin rash	53 (31.0)	41 (38.7)	12 (18.5)	0.006
Photosensitivity	13 (7.6)	10 (9.4)	3 (4.6)	0.374
Oral ulcer	21 (12.3)	19 (17.9)	2 (3.1)	0.004
Arthritis	10 (5.8)	8 (7.5)	2 (3.1)	0.322
Serositis	41 (24.0)	35 (33.0)	6 (9.2)	< 0.001
Neurologic disorder	2 (1.2)	1 (0.9)	1 (1.5)	0.726
Hematologic disorder	152 (88.9)	95 (89.6)	57 (87.7)	0.698
Immunologic disorder	159 (93.0)	100 (94.3)	59 (90.8)	0.376
**Comorbidities**				
Hypertension	33 (19.3)	11 (10.4)	22 (33.8)	< 0.001
Diabetes mellitus	4 (2.3)	3 (2.8)	1 (1.5)	0.999
Dyslipidemia	2 (1.2)	2 (1.9)	0 (0.0)	0.526

Values are expressed as median (interquartile range) or n (%)

*n/a* not applicable, *SLEDAI-2 K* systemic lupus erythematosus disease activity index-2000, *P/Cr* protein/creatinine, *WBC* white blood cell, *ESR* erythrocyte sedimentation rate, *CRP* C-reactive protein, *Cr* creatinine

**Table 2:** Comparison of baseline kidney histopathology data and prior immunosuppressive treatment

Variables	Total (*n* = 171)	Patients with early lupus nephritis (*n* = 106)	Patients with delayed lupus nephritis (*n* = 65)	*p*-value
**Kidney histopathology data**				
**Lupus nephritis class**				
Class I	3 (1.8)	0 (0.0)	3 (4.6)	0.053
Class II^a^	7 (4.1)	4 (3.8)	3 (4.6)	0.999
Class III^a^	57 (33.3)	35 (33.0)	22 (33.8)	0.801
Class IV^a^	84 (49.1)	53 (50.0)	31 (47.7)	0.770
Class V^a^	42 (24.6)	29 (27.4)	13 (20.0)	0.279
Class VI	0 (0.0)	0 (0.0)	0 (0.0)	0.999
**Activity/Chronicity**				
Activity index	7.0 (2.0–11.8)	7.0 (2.0–11.0)	8.0 (3.0–12.0)	0.323
Chronicity index, n (%)				0.007
0–1	104 (60.8)	71 (67.0)	33 (50.8)	
2–3	51 (29.8)	30 (28.3)	21 (32.3)	
4–5	13 (7.6)	4 (3.8)	9 (13.8)	
6	3 (1.8)	1 (0.9)	2 (3.1)	
**Prior immunosuppressive agent use**				
Glucocorticoids	64 (37.4)	0 (0.0)	64 (98.5)	< 0.001
Cyclophosphamide	1 (0.6)	0 (0.0)	1 (1.5)	0.380
Mycophenolate mofetil	2 (1.2)	0 (0.0)	2 (3.1)	0.143
Tacrolimus	1 (0.6)	0 (0.0)	1 (1.5)	0.380
Cyclosporine	2 (1.2)	0 (0.0)	2 (3.1)	0.143
Azathioprine	10 (5.8)	0 (0.0)	10 (15.4)	< 0.001
Methotrexate	3 (1.8)	0 (0.0)	3 (4.6)	0.053
Hydroxychloroquine	44 (25.7)	0 (0.0)	44 (67.7)	< 0.001

Values are expressed as median (interquartile range) or n (%)

^a^Mixed lupus nephritis cases were counted for each respective class

**Table 3:** Drugs administered during the follow-up

Variables	Total (*n* = 171)	Patients with early lupus nephritis (*n* = 106)	Patients with delayed lupus nephritis (*n* = 65)	*p*-value
**Immunosuppressive agents**				
Glucocorticoids	171 (100.0)	106 (100.0)	65 (100.0)	0.999
Cumulative glucocorticoid dosage (mg)^a^	11,315.0 (5195.3–18,697.5)	10,889.6 (5145.0–17,306.3)	14,370.0 (5472.5–20,703.8)	0.181
Cyclophosphamide	57 (33.3)	42 (39.6)	15 (23.1)	0.026
Mycophenolate mofetil	133 (77.8)	83 (78.3)	50 (76.9)	0.834
Tacrolimus	37 (21.6)	24 (22.6)	13 (20.0)	0.685
Cyclosporine	8 (4.7)	6 (5.7)	2 (3.1)	0.711
Azathioprine	24 (14.0)	13 (12.3)	11 (16.9)	0.396
Hydroxychloroquine	98 (57.3)	55 (51.9)	43 (66.2)	0.051

Values are expressed as median (interquartile range) or n (%)

^a^Cumulative glucocorticoid dosage was calculated in prednisolone equivalent dosage

### Comparison of clinical outcomes in patients with early and delayed lupus nephritis

**Fig. 1:**
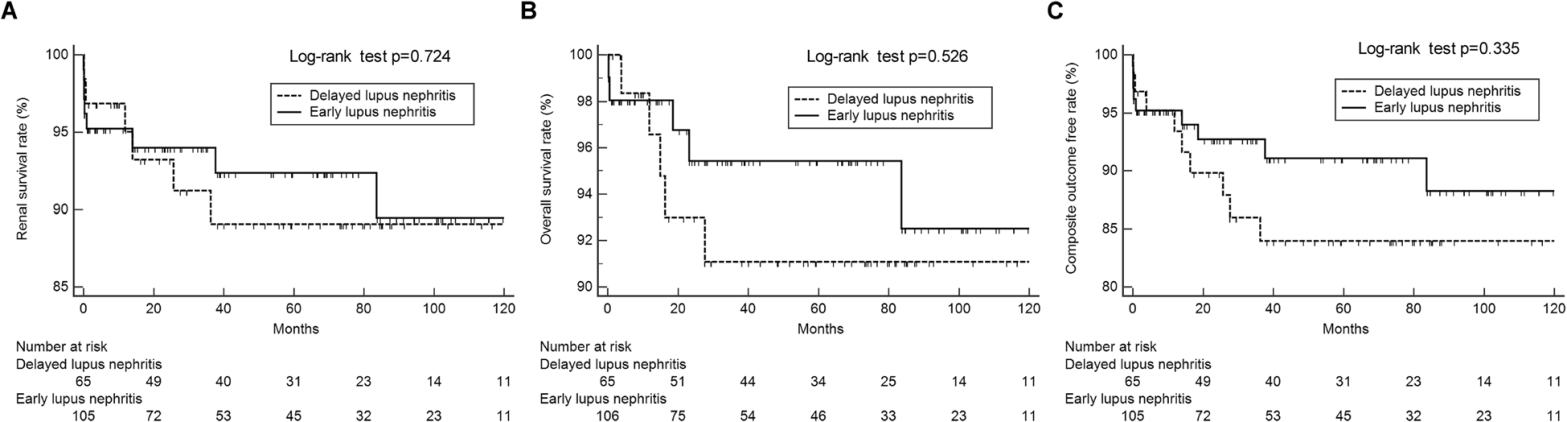
Kaplan-Meier curve analysis of clinical outcomes in patients with early and delayed lupus nephritis. The clinical outcomes of renal survival rate (**a**), overall survival rate (**b**), and composite outcome free rate (**c**) was compared in patients with early and delayed lupus nephritis

Kaplan-Meier analysis was carried out to compare the clinical outcomes in patients with early and delayed lupus nephritis. No differences in renal and overall survival rates were found between the groups (log-rank test *p* = 0.720 and *p* = 0.526, Fig. 1a–b). Moreover, the composite outcome free rate was also comparable between early and delayed lupus nephritis (log-rank test *p* = 0.335, Fig. 1c).

### Predictive factors of the development of ESRD in lupus nephritis

Among included variables, age, hemoglobin, CRP and creatinine levels and chronicity index revealed by renal biopsy were associated with the development of ESRD in univariate Cox-proportional hazard analysis. Multivariate analysis revealed that CRP level (odds ratio [OR] 1.021, 95% confidence interval [CI] 1.006–1.037, *p* = 0.007), creatinine level (OR 2.233, 95% CI 1.539–3.239, *p* < 0.001) and chronicity index (OR 1.475, 95% CI 1.042–2.090, *p* = 0.029) were predictive factors of ESRD (Table 4).

**Table 4:** Univariate and multivariate Cox-proportional hazard analysis of variables associated with end-stage renal disease

	Univariate analysis			Multivariate analysis		
	Odds ratio	95% CI	p-value	Odds ratio	95% CI	*p*-value
Age, years	1.046	1.007–1.086	0.021			
Female sex	1.648	0.215–12.638	0.631			
SLEDAI-2 K	0.915	0.791–1.057	0.227			
Complement 3, mg/dL	0.989	0.967–1.011	0.327			
Complement 4, mg/dL	0.971	0.897–1.051	0.470			
Anti-dsDNA (IU/mL)	0.998	0.995–1.001	0.183			
Spot urine P/Cr ratio	1.001	0.863–1.159	0.995			
WBC count (/μL)	1.000	0.999–1.000	0.900			
Platelet count (×1000/μL)	0.995	0.989–1.002	0.139			
Hemoglobin (g/dL)	0.601	0.455–0.795	< 0.001			
Lymphocyte count (/μL)	0.999	0.998–1.000	0.099			
ESR (mm/hr)	1.006	0.990–1.022	0.493			
CRP (mg/L)	1.015	1.002–1.028	0.028	1.021	1.006–1.037	0.007
Cr (mg/dL)	2.445	1.801–3.317	< 0.001	2.233	1.539–3.239	< 0.001
Albumin (g/dL)	0.620	0.306–1.259	0.186			
Activity index	1.058	0.959–1.167	0.261			
Chronicity index	1.581	1.199–2.083	0.001	1.475	1.042–2.090	0.029
Early lupus nephritis	0.830	0.295–2.337	0.725			
Delayed lupus nephritis	1.204	0.428–3.388	0.725			

*SLEDAI-2 K* systemic lupus erythematosus disease activity index-2000, *P/Cr* protein/creatinine, *WBC* white blood cell, *ESR* erythrocyte sedimentation rate, *CRP* C-reactive protein, *Cr* creatinine

### Predictive factors associated with all-cause mortality in lupus nephritis

The univariate Cox-proportional hazard analysis showed that age and hemoglobin and CRP levels were predictive of all-cause mortality. However, in multivariate analysis, only age (OR 1.065, 95% CI 1.018–1.114, *p* = 0.006) and hemoglobin level (OR 0.656, 95% CI 0.448–0.959, *p* = 0.030) were factors that were independently associated with all-cause mortality during the follow-up (Table 5).

**Table 5:** Univariate and multivariate Cox-proportional hazard analysis of variables associated with all-cause mortality

	Univariate analysis			Multivariate analysis		
	Odds ratio	95% CI	p-value	Odds ratio	95% CI	*p*-value
Age, years	1.066	1.019–1.115	0.006	1.065	1.018–1.114	0.006
Female sex	1.117	0.141–8.851	0.917			
SLEDAI-2 K	0.927	0.782–1.099	0.382			
Complement 3, mg/dL	0.990	0.963–1.017	0.443			
Complement 4, mg/dL	0.924	0.814–1.048	0.220			
Anti-dsDNA (IU/mL)	0.998	0.995–1.002	0.314			
Spot urine P/Cr ratio	0.682	0.458–1.017	0.060			
WBC count (/μL)	1.000	0.999–1.000	0.445			
Platelet count (×1000/μL)	1.000	0.993–1.007	0.996			
Hemoglobin (g/dL)	0.653	0.467–0.913	0.013	0.656	0.448–0.959	0.030
Lymphocyte count (/μL)	0.999	0.998–1.001	0.361			
ESR (mm/hr)	1.010	0.991–1.029	0.323			
CRP (mg/L)	1.017	1.001–1.033	0.034			
Cr (mg/dL)	1.479	0.889–2.461	0.132			
Albumin (g/dL)	0.927	0.399–2.152	0.860			
Activity index	1.004	0.892–1.129	0.954			
Chronicity index	1.233	0.861–1.766	0.252			
Early lupus nephritis	0.671	0.194–2.319	0.529			
Delayed lupus nephritis	1.490	0.431–5.148	0.529			
Hypertension	2.965	0.834–10.539	0.093			
Diabetes mellitus^a^	n/a					
Dyslipidemia^a^	n/a					

*SLEDAI-2 K* systemic lupus erythematosus disease activity index-2000, *P/Cr* protein/creatinine, *WBC* white blood cell, *ESR* erythrocyte sedimentation rate, *CRP* C-reactive protein, *Cr* creatinine, *n/a* not applicable

^a^The odds ratio was not calculable because no death occurred in patients with diabetes mellitus and dyslipidaemia

## Discussion

The results of this study showed that lupus nephritis affected more than 60% of patients at the time of SLE diagnosis; however, a substantial number of patients developed lupus nephritis after SLE was diagnosed. In this study, the median disease duration of SLE in the delayed lupus nephritis group was < 5 years, which is consistent with data of previous studies [8]. Regarding the clinical characteristics, when lupus nephritis developed early in the course of SLE, affected patients were more likely to have higher disease activity and prominent multiple organ involvement. This finding could be related to the fact that almost every patient with delayed lupus nephritis was being currently or previously treated with immunosuppressive agents. Therefore, it is more likely for patients with early lupus nephritis to demonstrate higher disease activity and systemic inflammation compared to those with delayed lupus nephritis. However, during the follow-up period, no difference was found in the clinical outcomes of the patients, probably due to the similar effect of administered treatment in these patients. Therefore, the observations from the current study provide useful information regarding the management of patients with lupus nephritis.

An important finding of our study was that the prognosis of patients with early and delayed lupus nephritis was not significantly different. This fact might be explained by several reasons. First, because current ACR guidelines recommend kidney biopsy for patients with SLE to assess the possibility of lupus nephritis when proteinuria exceeds 1 g [4], the timely intervention to diagnose lupus nephritis and manage inflammation might have hampered the development of irreversible organ damage and in affecting patient mortality. Thus, our findings further emphasize that strict adherence to the current practice guidelines is essential for the proper management of SLE. Second, only a few patients with delayed lupus nephritis were previously treated with cyclophosphamide and mycophenolate mofetil, which is the most commonly used immunosuppressive agent to induce remission in lupus nephritis [15]. Therefore, while almost every patient with delayed lupus nephritis were on immunosuppression, the prior treatment might not have been sufficient to prevent the development of lupus nephritis and influence the treatment response of induction and maintenance therapies. Conversely, although patients with early lupus nephritis exhibited higher disease activity, and thus, were more likely to be treated with cyclophosphamide, the application of aggressive treatment with cyclophosphamide might have resulted in similar clinical outcomes in early and delayed lupus nephritis.

Notably, a recent publication by Ugolini-Lopes and colleagues have evaluated the clinical outcomes in patients with early-onset and late-onset lupus nephritis and found a similar prognosis in both groups [10]. In their study, early-onset lupus nephritis was defined as lupus nephritis occurring in the first 5 years of SLE diagnosis and late-onset lupus nephritis as that occurring after 5 years of disease diagnosis. Similar findings were also found regarding the clinical outcomes in this study, and the renal and overall survival rate of early and delayed lupus nephritis was not significantly different in Kaplan-Meier curve analysis. Although different definitions were adopted for early and delayed lupus nephritis, the present study has several advantages compared to the study by Ugolini-Lopes et al. in terms of a larger number of patients with Asian ethnicity, evaluated factors associated with ESRD and all-cause mortality and detailed data regarding immunosuppressive agents used before and after the diagnosis of lupus nephritis.

Even though the advances of lupus nephritis treatment have led to a lower occurrence of ESRD in the recent decades [16], ESRD still remains to be one of the most morbid condition in lupus nephritis [17]. In multivariate Cox-proportional hazard analysis, higher creatinine and CRP levels along with higher chronicity index at baseline were found to be predictive factors for ESRD. Consistently, poor renal function at initial presentation and higher chronicity index were shown to be predictors of ESRD progression in patients with lupus nephritis [18]. An interesting finding of this study was that even though patients with delayed lupus nephritis had a greater proportion of patients with higher chronicity index, the renal outcome was not significantly different compared to those of patients with early lupus nephritis. This could be related to the fact that the difference in the chronicity index between the groups was not large enough to reach statistical significance. Therefore, this finding should be verified in future studies.

It has been reported that lupus nephritis is associated with significantly higher mortality in SLE [19]. When we evaluated factors associated with higher mortality, age was associated with increased risk of mortality, while higher hemoglobin level was associated with lower risk of mortality independently of traditional risk factors, such as of hypertension, diabetes mellitus and dyslipidaemia [20–22]. Old age is closely linked to increased risk of mortality in the general population [23]. However, the association with mortality in lupus nephritis is controversial. A previous study has demonstrated that age was a predictor of death in patients with lupus nephritis [24], while in a recent publication by Teh et al. age was not associated with the risk of mortality [25]. These discrepant results between the studies may be related to the different ethnic groups included and the selection of variables for the analysis. On the other hand, anemia was also reported to be an independent factor related to mortality in patients with chronic inflammatory diseases and malignancies [26–28]. Although the direct association between anemia and higher mortality in lupus nephritis is unclear, it could be indirectly associated with higher inflammation in lupus nephritis based on the fact that hemoglobin level could decrease in association with inflammatory burden or as a consequence of impaired renal function [29, 30]. Overall, it could be suggested that age and hemoglobin level should be taken into account when predicting mortality among patients with lupus nephritis.

The main strengths of the present study were the large number of included patients with Asian ethnicity and pathologically confirmed lupus nephritis for the evaluation of outcomes. However, it also has several inherent limitations. First, patient data and adverse effects of glucocorticoid and immunosuppressive agents were investigated retrospectively by reviewing the medical records. Second, the follow-up period of the included patients was relatively short. Third, we were not able to precisely assess the effect of used immunosuppressive agents on the patient prognosis. Fourth, the application of 1997 revised ACR classification criteria, which possess lower sensitivity as compared to the 2012 SLE International Collaborating Clinics criteria and the 2019 European League Again Rheumatism/ACR criteria, could have resulted in selection or classification bias. Additional investigations are necessary to comparatively assess clinical outcomes of early and delayed lupus nephritis.

## Conclusions

In conclusion, the distinct clinical features of early lupus nephritis are higher disease activity and more frequent multiple organ involvement. However, long-term clinical outcomes of early and delayed lupus nephritis appear to be similar. Rigorous adherence to current treatment recommendations is important in providing optimal treatment for patients with lupus nephritis.

## Supplementary information

**Additional file 1: Supplementary Table 1.** Comparison of adverse effects of glucocorticoids and immunosuppressive agents between patients with early and delayed lupus nephritis.

## Data Availability

The datasets used and/or analysed during the current study are available from the corresponding author on reasonable request.

## Abbreviations

ACR: American College of Rheumatology
C: Complement
CI: Confidence interval
CRP: C-reactive protein
DUR: Drug utilization review
ESRD: End-stage renal disease
ISN/RPS: International Society of Nephrology/ Renal Pathology Society
OR: Odds ratio
P/Cr: Protein/creatinine
SLE: Systemic lupus erythematosus
SLEDAI-2 K: SLE disease activity index-2000

## References

[1] Rahman A, Isenberg DA (2008). Systemic lupus erythematosus. N Engl J Med.

[2] Davidson A (2016). What is damaging the kidney in lupus nephritis?. Nat Rev Rheumatol.

[3] Markowitz GS, D'Agati VD (2007). The ISN/RPS 2003 classification of lupus nephritis: an assessment at 3 years. Kidney Int.

[4] Hahn BH, McMahon MA, Wilkinson A, Wallace WD, Daikh DI, Fitzgerald JD, Karpouzas GA, Merrill JT, Wallace DJ, Yazdany J (2012). American College of Rheumatology guidelines for screening, treatment, and management of lupus nephritis. Arthritis Care Res.

[5] Norby GE, Mjoen G, Bjorneklett R, Vikse BE, Holdaas H, Svarstad E, Aasarod K (2017). Outcome in biopsy-proven lupus nephritis: evaluation of biopsies from the Norwegian kidney biopsy registry. Lupus.

[6] Wilhelmus S, Bajema IM, Bertsias GK, Boumpas DT, Gordon C, Lightstone L, Tesar V, Jayne DR (2016). Lupus nephritis management guidelines compared. Nephrol Dial Transplant.

[7] Seligman VA, Lum RF, Olson JL, Li H, Criswell LA (2002). Demographic differences in the development of lupus nephritis: a retrospective analysis. Am J Med.

[8] Alexandre AR, Carreira PL, Isenberg DA (2018). Very delayed lupus nephritis: a report of three cases and literature review. Lupus Sci Med.

[9] Gordon C, Amissah-Arthur MB, Gayed M, Brown S, Bruce IN, D'Cruz D, Empson B, Griffiths B, Jayne D, Khamashta M (2018). The British Society for Rheumatology guideline for the management of systemic lupus erythematosus in adults. Rheumatology (Oxford).

[10] Ugolini-Lopes MR, Santos LPS, Stagnaro C, Seguro LPC, Mosca M, Bonfa E (2019). Late-onset biopsy-proven lupus nephritis without other associated autoimmune diseases: severity and long-term outcome. Lupus.

[11] Hochberg MC (1997). Updating the American College of Rheumatology revised criteria for the classification of systemic lupus erythematosus. Arthritis Rheum.

[12] Kim SK, Choe JY, Lee SS (2018). Self-reported physical activity is associated with lupus nephritis in systemic lupus erythematosus: data from KORean lupus network (KORNET) registry. Yonsei Med J.

[13] Gladman DD, Ibanez D, Urowitz MB (2002). Systemic lupus erythematosus disease activity index 2000. J Rheumatol.

[14] Austin HA, Boumpas DT, Vaughan EM, Balow JE (1994). Predicting renal outcomes in severe lupus nephritis: contributions of clinical and histologic data. Kidney Int.

[15] Yu F, Haas M, Glassock R, Zhao MH (2017). Redefining lupus nephritis: clinical implications of pathophysiologic subtypes. Nat Rev Nephrol.

[16] Tektonidou MG, Dasgupta A, Ward MM (2016). Risk of End-Stage Renal Disease in Patients With Lupus Nephritis, 1971-2015: A systematic review and Bayesian meta-analysis. Arthritis Rheumatol (Hoboken).

[17] Lateef A, Petri M (2012). Unmet medical needs in systemic lupus erythematosus. Arthritis Res Ther.

[18] Contreras G, Pardo V, Cely C, Borja E, Hurtado A, De La Cuesta C, Iqbal K, Lenz O, Asif A, Nahar N (2005). Factors associated with poor outcomes in patients with lupus nephritis. Lupus.

[19] Yap DY, Tang CS, Ma MK, Lam MF, Chan TM (2012). Survival analysis and causes of mortality in patients with lupus nephritis. Nephrol Dial Transpl.

[20] Zhou D, Xi B, Zhao M, Wang L, Veeranki SP (2018). Uncontrolled hypertension increases risk of all-cause and cardiovascular disease mortality in US adults: the NHANES III linked mortality study. Sci Rep.

[21] Rao Kondapally Seshasai S, Kaptoge S, Thompson A, Di Angelantonio E, Gao P, Sarwar N, Whincup PH, Mukamal KJ, Gillum RF, Holme I (2011). Diabetes mellitus, fasting glucose, and risk of cause-specific death. New Engl J Med.

[22] Murray CJ, Atkinson C, Bhalla K, Birbeck G, Burstein R, Chou D, Dellavalle R, Danaei G, Ezzati M, Fahimi A (2013). The state of US health, 1990-2010: burden of diseases, injuries, and risk factors. JAMA.

[23] Ahn C, Hwang Y, Park SK (2017). Predictors of all-cause mortality among 514,866 participants from the Korean National Health Screening Cohort. PLoS One.

[24] Faurschou M, Dreyer L, Kamper AL, Starklint H, Jacobsen S (2010). Long-term mortality and renal outcome in a cohort of 100 patients with lupus nephritis. Arthritis Care Res.

[25] Teh CL, Phui VE, Ling GR, Ngu LS, Wan SA, Tan CH (2018). Causes and predictors of mortality in biopsy-proven lupus nephritis: the Sarawak experience. Clin Kidney J.

[26] Dicato M, Plawny L, Diederich M (2010). Anemia in cancer. Ann Oncol.

[27] Madu AJ, Ughasoro MD (2017). Anaemia of chronic disease: an in-depth review. Med Princ Pract.

[28] Weiss G, Ganz T, Goodnough LT (2019). Anemia of inflammation. Blood.

[29] Nemeth E, Ganz T (2014). Anemia of inflammation. Hematol Oncol Clin North Am.

[30] Babitt JL, Lin HY (2012). Mechanisms of anemia in CKD. J Am Soc Nephrol.

