# Supplementary material for: Comparison of clinical features and outcomes between patients with early and delayed lupus nephritis
Source: BMC Nephrol. 2020 Jul 7;21:258. doi: 10.1186/s12882-020-01915-5 (PMC7341643; doi:10.1186/s12882-020-01915-5)
Supplement: Supplementary file 1 — Additional file 1: Supplementary Table 1. Comparison of adverse effects of glucocorticoids and immunosuppressive agents between patients with early and delayed lupus nephritis. [file 12882_2020_1915_MOESM1_ESM.docx]

**Supplementary Table 1. Comparison of adverse effects of glucocorticoids and immunosuppressive agents between patients with early and delayed lupus nephritis**

| Adverse effects | Patients with  early lupus nephritis (n=106) | Patients with delayed lupus nephritis (n=65) | p-value |
| --- | --- | --- | --- |
| **Infections** |  |  |  |
| Herpes zoster | 9 (8.5) | 4 (6.2) | 0.768 |
| Other | 34 (32.1) | 29 (44.6) | 0.100 |
| **Systemic effects** |  |  |  |
| Weight gain | 16 (15.1) | 9 (13.8) | 0.823 |
| Facial edema | 23 (21.7) | 19 (29.2) | 0.268 |
| Generalized edema | 26 (24.5) | 13 (20.0) | 0.495 |
| Hyperglycemia | 4 (3.8) | 2 (3.1) | 0.999 |
| **Dermatologic effects** |  |  |  |
| Acne | 6 (5.7) | 5 (7.7) | 0.600 |
| Bruising | 3 (2.8) | 1 (1.5) | 0.999 |
| **Gastrointestinal effects** |  |  |  |
| Gastrointestinal discomfort | 15 (14.2) | 7 (10.8) | 0.523 |
| **Musculoskeletal effects** |  |  |  |
| Myalgia | 8 (7.5) | 0 (0.0) | 0.025 |
| Avascular necrosis | 1 (0.9) | 1 (1.5) | 0.999 |
| **Nervous system effects** |  |  |  |
| Insomnia | 13 (12.3) | 8 (12.3) | 0.993 |
| Depression | 2 (1.9) | 1 (1.5) | 0.999 |
| Psychosis | 2 (1.9) | 0 (0.0) | 0.526 |
| **Ophthalmologic effects** |  |  |  |
| Central serous chorioretinopathy | 1 (0.9) | 3 (4.6) | 0.154 |
| Cataract | 0 (0.0) | 2 (3.1) | 0.143 |
| Glaucoma | 1 (0.9) | 0 (0.0) | 0.999 |
| **None** | 30 (28.3) | 15 (23.1) | 0.453 |

Values are expressed as n (%).
